# Bio-based Polyurethane Composites from Macaúba Kernel Oil: Part 2, Wood Thermal Treatment and Composite Water Sorption Properties

**DOI:** 10.1021/acsomega.6c04518

**Published:** 2026-07-10

**Authors:** Rodolfo Andrade Breves, Roseany de Vasconcelos Vieira Lopes, Rafael Lopes Quirino, Baptiste Colin, Anelie Petrissans, Maria José Araujo Sales, Mathieu Petrissans

**Affiliations:** † 28127 University of Brasília, Chemistry Institute, LabPolN, Campus Universitario Darcy Ribeiro, Brasilia, Distrito Federal 70910-000, Brazil; ‡ IUT Hubert Curien, LERMAB, INRAE, Université de Lorraine, 7, Rue Fusilles de la Resistance, Epinal 88000, France; § University of Brasília, Campus Gama, Gama, Distrito Federal 72429-005, Brazil; ∥ Center for Advanced Materials Science, Department of Biochemistry, Chemistry and Physics, 123388 Georgia Southern University, P.O. Box 8064, Statesboro, Georgia 30460, United States

## Abstract

Polymer composites are the result of reinforcing a polymeric matrix with other materials. For example, the reinforcement of polyurethanes (PU) with natural fibers is a popular combination because of the great chemical compatibility between the two materials. This work investigates the production of composites using a polyurethane matrix synthesized from macaúba kernel oil and glycerol, reinforced with beech, fir, and carvoeiro. A thermal treatment was applied to the wood reinforcements, and the impact of the thermal treatment on chemical, thermal, and water sorption properties was extensively investigated. The composites prepared exhibited negligible differences in Fourier transform infrared (FTIR) and thermogravimetric analysis (TGA) when compared to the starting PU, showing that the addition of both thermally treated and untreated wood resulted in similar products, with comparable functional groups and thermal stabilities. However, the addition of 2 wt % of thermally treated wood drastically influences the water sorption properties and maximum water uptake under DVS, reaching values several times higher than the unreinforced samples.

## Graphical abstract

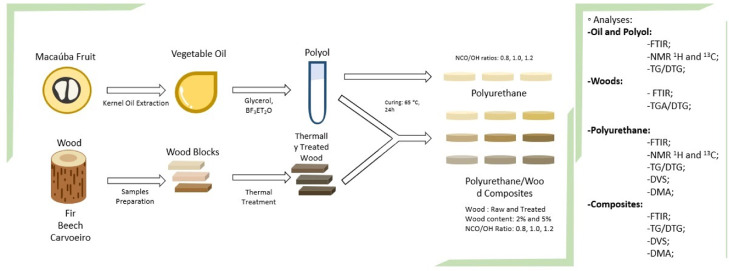

## Introduction

This article is the second part of a study on the development of biocomposites made from polyurethane (PU) produced from macaúba kernel oil (MKO) and glycerol.^1^ While the first part focused on the production of the PU itself, the current manuscript discusses the production of bio-based composites, their properties, and the effect of wood thermal treatment on the properties of the composites.

Composites are materials made up of at least two different components with their own boundaries and properties, such as PU and natural fibers. Most materials produced nowadays can be considered composites.^2,3^ Natural fibers show great potential for numerous applications; they are available in great varieties, large quantities, and are biodegradable.^3^ Composites made with them show good potential for application in insulation, for example, as demonstrated by the works of Azzouzi et al. (2020),^4^ who created composites by combining plaster and ground pea pods, resulting in materials with better insulating properties than commercial products. Natural fibers show great compatibility with PU matrices. PUs are formed by the reaction of polyols with diisocyanates (OCN–R–NCO, where R is an organic moiety),^5^ while natural fibers are composed mostly of hemicelluloses, cellulose, and lignin. Since the fiber components are highly hydroxylated, the isocyanate groups (−NCO) can react with them, as well as with the hydroxyl groups of the polyol used in the PU formulation.^6–8^ Figure 1 shows the reaction between an isocyanate functional group (−NCO) and a polyol.

**Figure 1:**
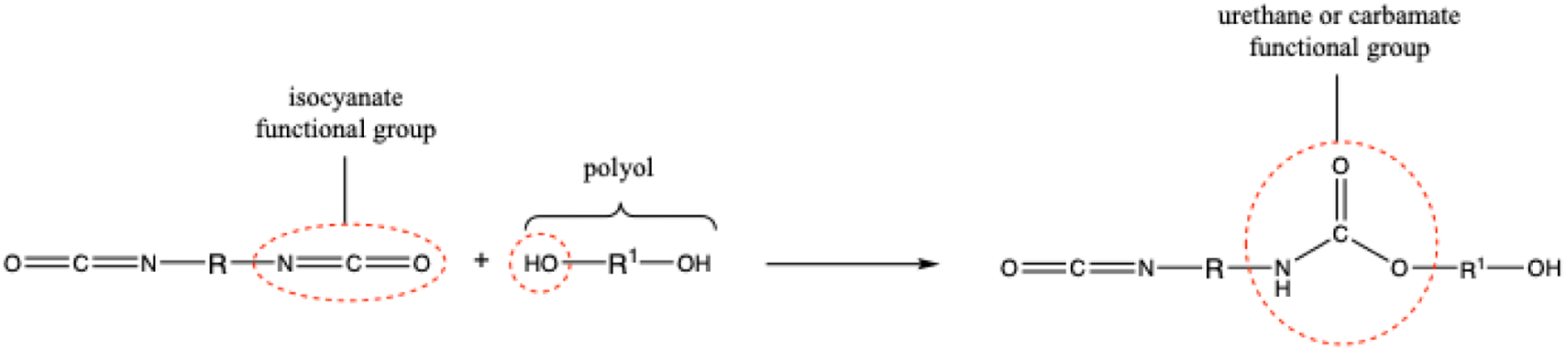
Formation of a urethane (or carbamate) functional group from the reaction between an isocyanate and a hydroxyl group of a polyol. “R” and “R^1^” represent aliphatic or aromatic chains. Original drawing by the authors.

The compatibility between polyurethanes and natural fibers has led researchers, such as Lopes et al. (2021),^9^ to develop composite strand boards made from castor oil-based PU and bamboo fiber residue, with loadings ranging from 20 vol % to 40 vol %. Their products showed good moisture absorption and mechanical and flexural strength and, overall, met the commercial standards for strand boards.^9^ Hemicelluloses are a family of polysaccharides composed of different sugars, resulting in short and branched chains, while cellulose is made entirely of cellobiose (glucose dimers) with high degrees of polymerization and long, linear chains. This structure makes cellulose a mechanically stronger polymer than hemicelluloses.^6,10^ Figure 2 shows the chemical structures of an example of a hemicellulose (xylan) and cellulose.

**Figure 2:**
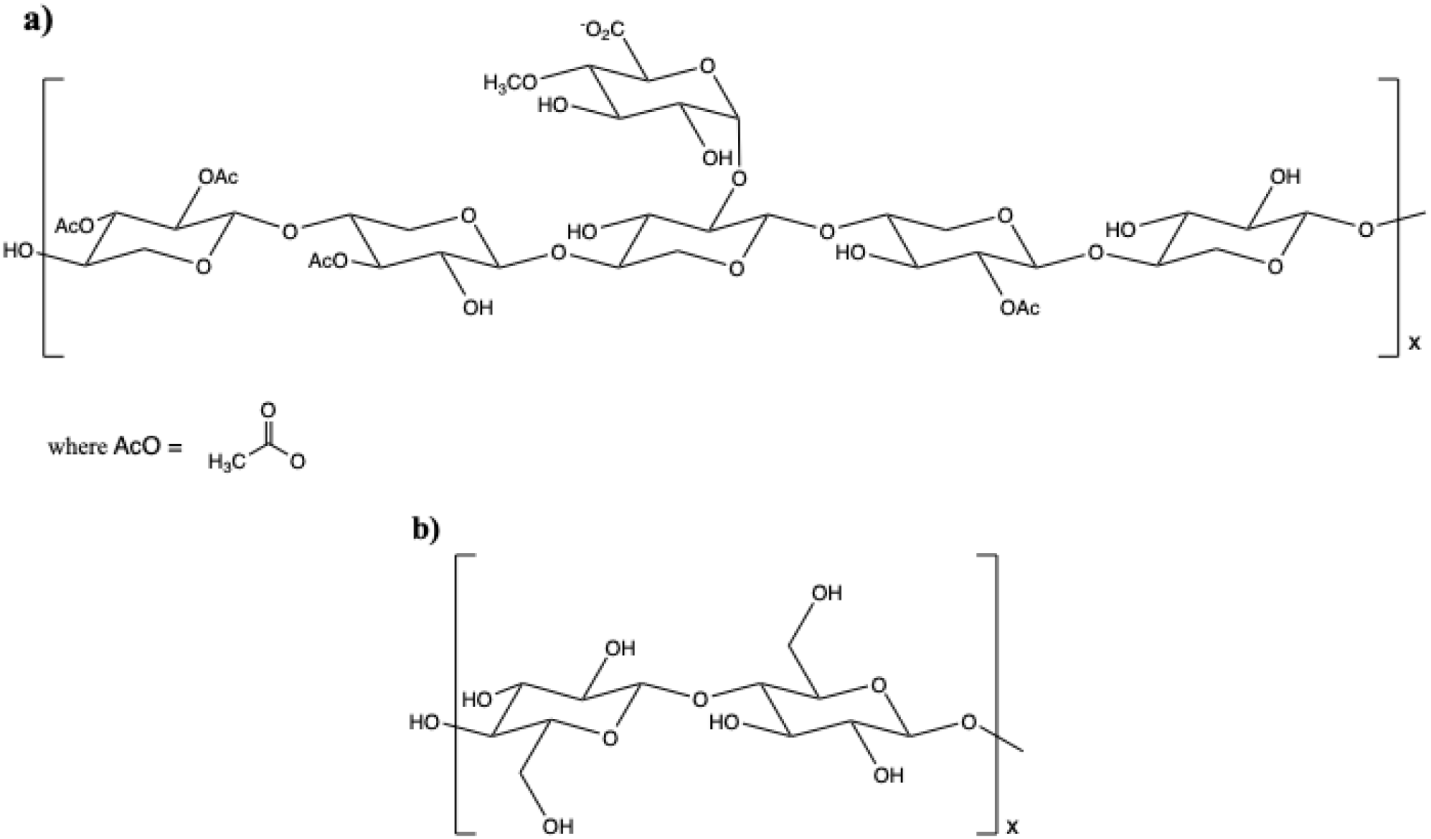
Chemical structures of (a) xylan, a hemicellulose, and (b) cellulose. Original drawing by the authors.

As cellulose is a stronger polymer than hemicelluloses, it is advantageous to remove hemicelluloses from natural fibers so that the PU formation can occur primarily with cellulose. There are various ways for hemicellulose removal. One example is alkaline treatment,^3,11^ although it leads to partial degradation of cellulose.^12^ Thermal treatments are common in the wood industry. They are used to partially degrade the less thermally stable components of the wood, such as hemicelluloses.^13–16^ Studies reported in the literature demonstrate that thermal treatments can improve some of the properties of the wood while compromising others.

While thermal treatments are quite complex, changes in wood composition can be broadly understood by careful analysis of the derivative of a TGA curve (DTG). Indeed, when a ligno-cellulosic sample is submitted to a temperature ramp from room temperature to 700 °C, at a constant heating rate, in TGA, the resulting derivative curve reveals features that can be linked to specific wood components (hemicelluloses, cellulose, lignin). By deconvoluting these curves and integrating each peak, it is possible to quantify individual wood components in a convenient way, as demonstrated by Carrier et al. (2011).^17^ As most hemicelluloses degrade at a lower temperature than crystalline cellulose,^1^ they can be selectively degraded. It is worth noting that a partial overlap can occur between amorphous cellulose and the more thermally resistant hemicelluloses, therefore, careful temperature control is critical to achieve the intended selective degradation of hemicelluloses in wood. Because hemicelluosesconstitute a family of biopolymers, each hemicellulose structure degrades at different temperature. Collectively, they exhibit a wide degradation peak on the DTG with low intensity, while crystalline cellulose exhibits a sharp and intense degradation peak.^17^ These features help identify the point at which the degradation of cellulose begins by observing what temperature causes a sharp increase in the sample’s mass loss.^1^

This study investigates the thermal degradation of three wood species, namely, (1) beech (*Fagus sylvatica*), a European hardwood; (2) fir (*Albies alba* *)*, a European softwood; and (3) carvoeiro (CV, *Tachigali vulgaris*), an understudied Brazilian wood that shows properties of hardwoods, like the presence of vessels and higher density.^18^ The objective is to selectively degrade the hemicelluloses without greatly affecting cellulose, followed by the application of both untreated and thermally treated woods as reinforcement materials in bio-based PUs prepared from macaúba kernel oil and glycerol. This is accomplished by the use of different indirect analyses (mass loss, DTG deconvolution, and delignification gravimetry) to follow the thermal degradation of the biopolymers of the wood. After establishing the appropriate treatment temperature, the treated woods were ground and used to prepare composites with 2 and 5 wt % of wood (both untreated and treated) while systematically varying the NCO:OH ratio of the PU from 0.8 to 1.2.^5^ The composites were finally analyzed by FTIR, TGA/DTG, and dynamic vapor sorption (DVS) to assess how the thermal treatment affects the chemical, thermal, and water sorption properties of the PU composites.

## Materials and Methods

### Materials

The samples of beech and fir were purchased from Vosges Promobois (Epinal, France), while CV samples were donated by Água Limpa Farm, a part of the Forestry Engineering Department at the University of Brasília (Brazil). All wood samples were cut into 14.0 cm × 6.0 cm × 2.0 cm (length × width × thickness) boards and dried at 103 °C for 24 h. After drying, the samples were stored in a desiccator at room temperature. The samples were weighted to determine their dry mass. Wood density for raw samples was calculated based on the dimensions of the boards and the dry mass determined. Only one measurement (mass/volume) was taken for each sample. The density was calculated only for raw samples, as the structural distortions caused by the thermal treatment compromised accurate dimension measurements.

Sodium chlorite (80% purity) was obtained from Thermo Scientific (Illkirch-Graffenstaden, France). Glacial acetic acid was obtained from Merck (Lyon, France), and sodium hydroxide was obtained from VWR (Fontenay-sous-Bois, France). Macaúba kernel oil (MKO) was purchased from Central do Cerrado (Brasília, Brazil), while formic acid 85%, hydrogen peroxide, and sodium carbonate were procured from Dinânimca (São Paulo, Brazil). Glycerol was procured from Labsynth (Diadema, Brazil), and boron trifluoride etherate (BF_3_·OEt_2_) was acquired from Fluka (Darmstadt, Germany). 4,4′-Diphenylmethane diisocyanate (MDI) was procured from Dow Chemical (São Paulo, Brazil), and sodium chloride (Cisne brand) was purchased from a local grocery store in Brazil.

### Thermal Treatment

The wood boards were placed, in pairs, inside a custom-built metal reactor (Figure 3). The system was initially flushed with N_2_ at room temperature with a flow rate of 25 mL·min^–1^ to eliminate any trace of oxygen. The nitrogen flow was kept constant during the treatment. The reactor was placed inside a convection oven and the temperature was increased at 2 °C·min^–1^ until the preset target, followed by a 30 min isothermal. After the treatment, the samples were cooled down to room temperature inside a desiccator, and their final mass was determined. The treated samples were stored at 103 °C. Treated and untreated samples of wood were ground and sieved into 3 different particle sizes, namely, <250 μm, 250–500 μm, and >500 μm. All ground samples were stored at 103 °C.

**Figure 3:**
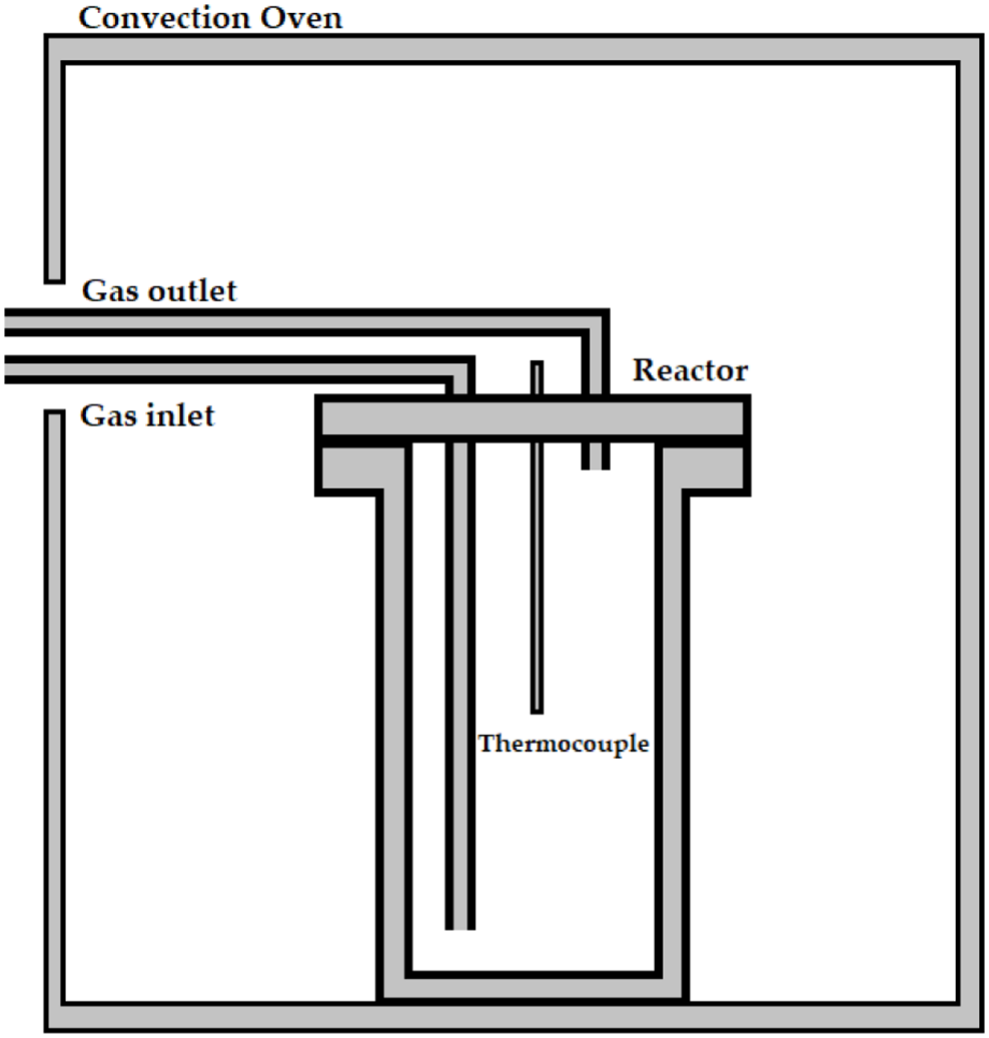
Schematic of the reactor used in the thermal treatments.

### Thermogravimetric Analyses (TGA)

Two different thermogravimetric profiles were used in this work (1) to quantify the biopolymers present in the wood before and after the thermal treatment and (2) to measure the thermal stability of the composites. A Mettler Toledo TGA 2 Stare (Greifensee, Switzerland) system was used to quantify the biopolymers before and after each thermal treatment. For TGA and DTG, the method used was adapted from Carrier et al. (2011),^17^ who quantified hemicelluloses and cellulose in several types of biomass. In the present study, 5.0–10.0 mg of ground wood was loaded onto alumina crucibles, and the temperature was increased at 5 °C·min^–1^ to 700 °C under a N_2_ flow rate of 100 mL·min^–1^. The TGA experiments used to measure the thermal stability of the composites were conducted on a Netzsch STA 449F3 (Selb, Germany) instrument. The temperature profile includes heating the sample from room temperature to 105 °C, followed by a 30 min isotherm. After that, the sample is heated to 700 °C. The experiments were conducted with a heating rate of 5 °C·min^–1^, under N_2_, at a flow rate of 33 mL·min^–1^.

Deconvolution of the DTG curves of the thermally treated samples was used to quantify the remaining hemicelluloses in each sample, adapting a previously published methodology.^17^ From the deconvoluted data, the hemicellulose and cellulose peaks were integrated, and the integrated values were used to calculate the cellulose:hemicellulose ratio (Cel:Hemi ratio). As the treatment temperature increases, the hemicelluloses degrade to a higher extent, decreasing their corresponding peak, while cellulose suffers little to no change. At the point where cellulose begins degrading, the cellulose integration value and, as a consequence, the Cel:Hemi ratio suffer a sharp decline, indicating the maximum temperature at which the wood can be treated while limiting cellulose degradation.

### Delignification Gravimetry

Delignification of wood samples was carried out following an adapted methodology from the literature.^19^ Ground wood samples underwent Soxhlet extraction with acetone overnight to remove low molecular weight extractives. Following extraction, the samples were dried at 103 °C for 24 h. After drying, approximately 4.0 g of ground wood was added to an Erlenmeyer flask with 160 mL of distilled water at room temperature. 1.6 g of sodium chlorite and 1.6 mL of acetic acid were added to the Erlenmeyer flask. The mixture was stirred at 70 °C for 8 h, with additions of 1.6 g of sodium chlorite and 1.6 mL of acetic acid every 2 h. Once all additions were completed, the mixture was vacuum filtered, and the solid product retained on the filter (holocellulose, consisting of a mixture of cellulose and hemicelluloses) was washed with distilled water until a neutral pH was obtained. The holocellulose was then carefully scraped from the filter, dried at 103 °C for 24 h, weighed, and finally stored in a desiccator.

### Fourier Transform Infrared (FTIR) Spectroscopy

FTIR was carried out on a Varian 640 (Jundiai, Brazil) spectrometer equipped with an Attenuated Total Reflectance (ATR) accessory. A spectral window from 4000 cm^–1^ to 600 cm^–1^ was used, and a total of 16 scans were collected per sample, with a spectral resolution of 4 cm^–1^. For comparison purposes, the spectra were normalized based on the H–C­(*sp* ^3^) signal at approximately 2900 cm^–1^.

### PU Synthesis and Composite Preparation

The PUs used as the polymeric matrix for the composites developed in this study were extensively discussed by the authors elsewhere.^5^ NCO:OH ratios of 0.8, 1.0, and 1.2 were used during PU synthesis. Macaúba kernel oil (MKO) was initially mixed with formic acid at room temperature. H_2_O_2_ was added dropwise under mechanical stirring. The mixture was then heated in a glycerin bath at 65 °C under agitation for 2 h to produce epoxidized MKO, which was isolated using a separatory funnel. The epoxidized oil was washed twice with a saturated NaCl solution and then neutralized with a 5% (m/v) Na_2_CO_3_ solution. The final product was dried in a rotary evaporator. Epoxidized MKO was then reacted with glycerol and BF_3_ etherate in diethyl ether for 6 h under agitation at room temperature. After the reaction, the polyol product was decanted in a separatory funnel, neutralized with a 10% (m/v) NaOH solution, and washed twice with a saturated NaCl solution. The appropriate quantities of polyol and MDI were added to a beaker and stirred for 1 min. The mixture was then transferred to round PTFE molds and cured in a preheated oven at 65 °C for 24 h. After curing, the molds were removed from the oven and left to cool down to room temperature before demolding.

Composites containing 2.0 and 5.0 wt % of raw and treated wood were used for each NCO:OH ratio. The composite synthesis followed the same steps as those described for PU synthesis. After the initial 1 min of stirring of polyol and diisocyanate, the wood particles were added, and the mixture was stirred for another minute. Curing and demolding procedures for composite preparation were exactly the same as those described for the PU synthesis.

### Dynamic Vapor Sorption (DVS)

DVS analyses were run on a DVS Intrinsic Plus instrument (Surface Measurement Systems, Heidelberg, Germany). Samples of approximately 5.0 mg of the composites were added to the scale in the humidity chamber, which was then sealed. The relative humidity in the chamber was raised from 0% to 95% in 5% increments and then decreased from 95% to 0% at the same rate. Mass stabilization was attained before each increment.

## Results and Discussion

### Wood Density

The results of wood density of the three studied species are listed in Table 1. It was verified that between the European woods, beech (a hardwood) has a higher density than fir (a softwood), as is typically the case. As for CV, the density is considerably higher than that of both European woods. However, it is worth pointing out that some of the CV’s properties are more similar to those of fir than beech, as will be discussed deeper later on.

**Table 1:** Density of Untreated Wood Species

Wood species	Density (kg·m^–3^)
Fir	519.21
Beech	673.92
CV	782.84

### Thermal Treatments

Figure 4 presents the results of mass loss after thermal treatment for the three wood species studied. From this point onward, the samples will be identified by a prefix representing the wood species (i.e., B for beech, F for fir, and CV for carvoeiro) and the treatment temperature (i.e., B270 is a beech sample treated at 270 °C).

**Figure 4:**
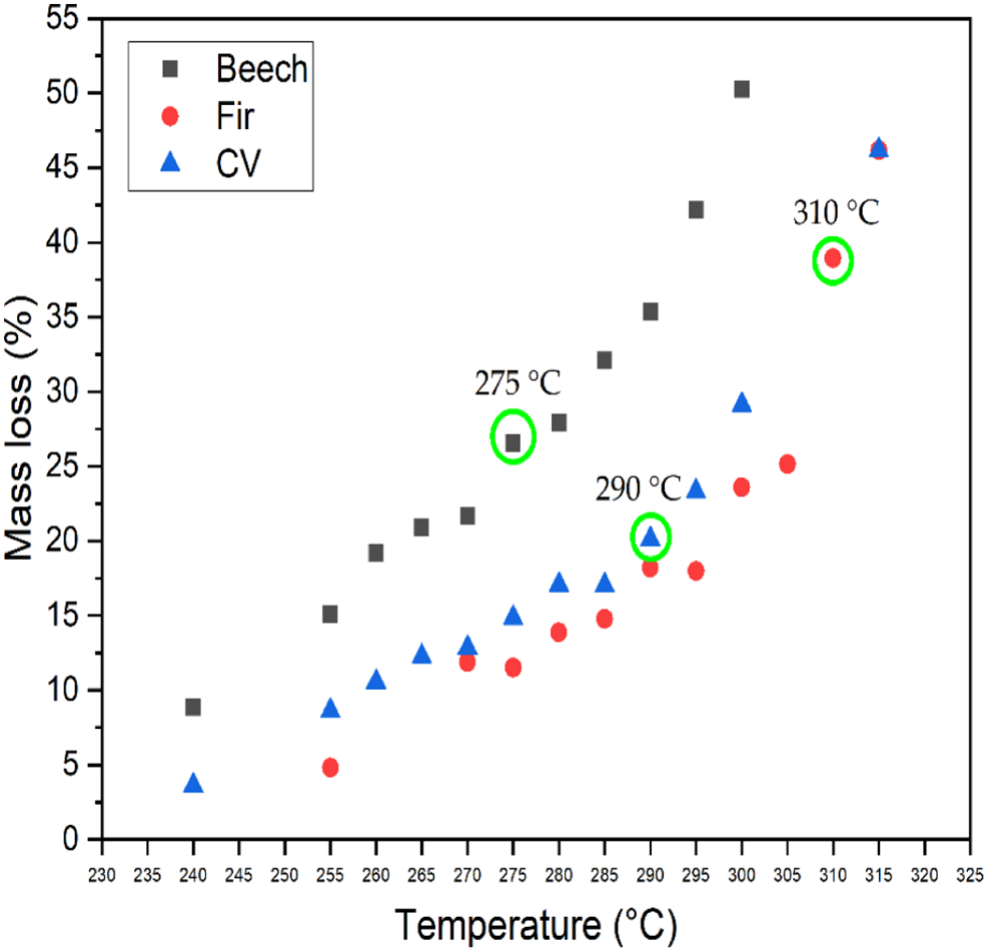
Mass loss after thermal treatment at different temperatures for beech, fir, and CV.

Figure 4 shows that despite its hardwood properties, the mass loss values of CV are much closer to fir than to beech. This could be an evolutionary adaptation of CV to survive in its biome, the Brazilian Cerrado, which has seasonal fires as part of its life cycle, making CV more thermally resistant than the European woods.

The objective of the thermal treatment experiments is to find a temperature in which the mass loss sharply increases, indicating the beginning of cellulose degradation. For beech, it is easy to identify this point at 275 °C, while for the other species, it was not as clear. A relatively large increase in mass loss is seen at 290 °C for CV. Also, in both beech and CV, the degradation rate is higher past the highlighted point, indicating degradation of cellulose. For fir, this point is harder to pinpoint. The mass loss value does not increase smoothly with the temperature increase, which could be caused by softwoods, differently from hardwoods, not having self-catalyzed hemicellulose thermal degradation,^20^ making each kind of hemicellulose degrade individually at their own temperatures. 310 °C was chosen as its increase in mass loss is much higher than what is seen in any other temperature increase. Following the same logic adopted for the ideal treatment temperature selection for both beech and CV, it is possible that fir treated at 315 °C could display an even greater mass loss than 310 °C, but considering a mass loss above 40 wt % implies the material integrity would be significantly compromised. Therefore, a treatment temperature of 310 °C was selected for fir.

From Figure 4, it can also be seen that beech and CV (both hardwoods) have higher mass loss values than fir (a softwood) at all temperatures. This is due to not only the degradation of hardwood hemicelluloses being self-catalyzed but also because hardwoods are naturally richer in hemicelluloses than softwoods,^21^ meaning they have more biomass available for degradation at lower temperatures. Ruxanda et al. (2008)^22^ and Mária et al. (2013)^21^ have quantified the hemicelluloses content of beech as 21.35 and 26.59 wt %, respectively. The temperature indicated in Figure 4 as the ideal thermal treatment temperature of beech (275 °C) corresponds to a mass loss of 24.41 wt %, indicating it is possibly a good temperature for the complete degradation of hemicelluloses.

For fir, Kučerová et al. (2019)^23^ and Senila et al. (2020)^24^ quantified the hemicellulose content to be 20 and 27 wt %, respectively. This means that the ideal temperature for the objective of this work would be between 305 and 310 °C. 310 °C was chosen due to the sharp increase in mass loss observed (Figure 4). Similar information regarding the hemicellulose content in CV could not be found in the literature.

### DTG Deconvolutions

DTG deconvolution was an additional method used to quantify the hemicellulose content left after thermal treatment of wood samples. The thermal decomposition of a wood sample can be broken down into five main steps, namely, (1) loss of water (<100 °C), (2) degradation of hemicelluloses (250–350 °C), (3) cellulose degradation (300–390 °C, with some overlap with (2)), (4) degradation of lignin (200–600 °C broad halo), and (5) degradation of other components, which can be byproducts of the wood thermal degradation itself. All of these steps are indicated in Figure 5, which indicates the change in composition as the treatment temperature for beech increases. A continuous relative decrease can be seen in the hemicellulose peak with respect to cellulose and lignin, as similarly observed by Carrier et al.^17^

**Figure 5:**
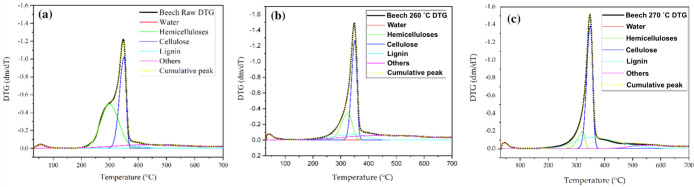
Deconvoluted DTG curves for (a) untreated beech and beech treated at (b) 260 °C and (c) 270 °C. Table S1 contains the % area under each deconvoluted curve.

Figure 6 represents the Cel:Hemi ratio for the deconvolutions of all samples. It provides a closer look at the thermodegradation behavior of the three studied wood species. For beech, there is a small but steady increase in the Cel:Hemi ratio, indicating that the degradation of hemicelluloses slowly increases with the treatment temperature up to 260 °C. Past this point, an intense degradation of hemicelluloses is seen up to 270 °C, followed by a drop in the ratio at 275 °C. This drop indicates the beginning of cellulose thermodegradation and sets the maximum treatment temperature for beech considering the objectives of this work. Beyond 275 °C, the deconvolution methodology employed did not result in satisfactory fitting, showing that, at that point, the hemicelluloses peak was either too small or simply no longer present, from which it can be inferred that the hemicelluloses have been completely degraded. The agreement between the results in Figures 5 and 6, as well as the literature^21,22^ shows consistency in the methodology employed in this work and confirms 275 °C as the ideal thermal treatment temperature for the selective degradation of hemicelluloses in beech.

**Figure 6:**
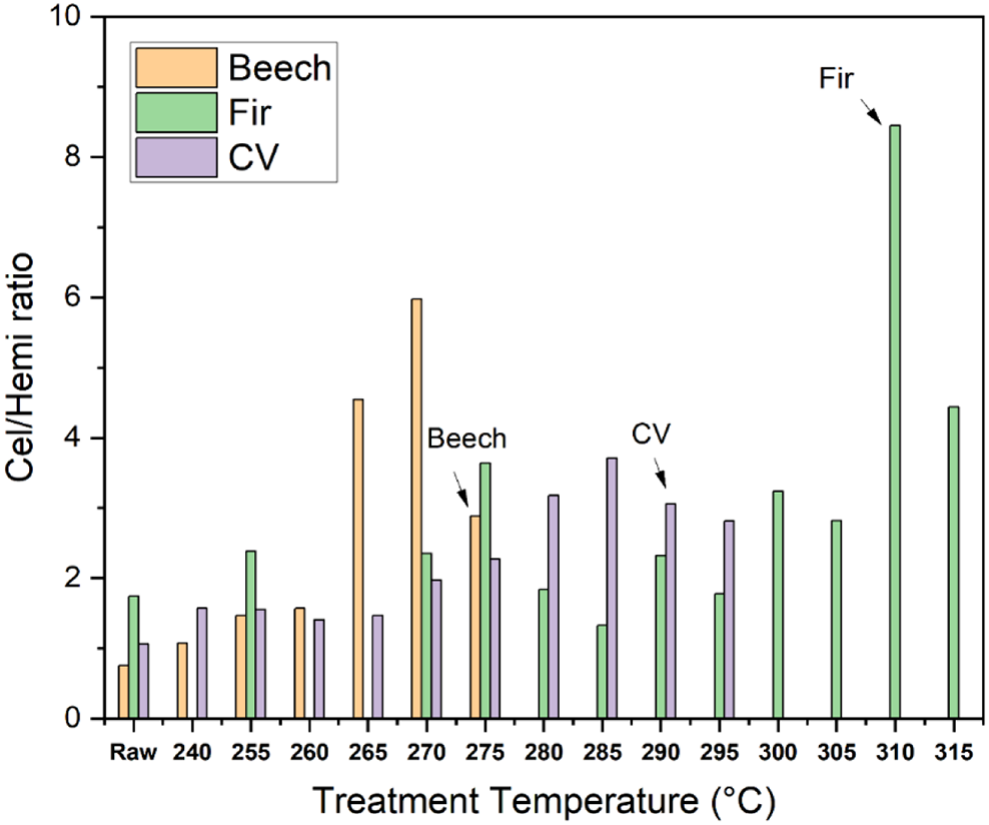
Cel:Hemi ratios calculated from the integration of deconvoluted peaks from the DTG of beech, fir, and CV.

CV (a hardwood) exhibits a similar behavior to beech, with stable Cel:Hemi values up to 265 °C, followed by a steady increase (although not as sharp as seen for beech), peaking at 285 °C, followed by a decrease at 290 °C, which indicates cellulose degradation. Following the same reasoning applied to beech, the appropriate temperature for the selective thermal degradation of hemicelluloses in CV seems to be 290 °C, which is also consistent with the mass loss results shown in Figure 4.

Unlike the hardwoods beech and CV, fir has not shown a steady increase to a maximum Cel:Hemi value. That could be due to the nature of the hemicelluloses in the softwood, as explained earlier in the text. Further studies that fall outside the intended scope of this work would be needed to confirm that. Here, the treatment temperature for which the Cel:Hemi ratio drops is 315 °C. This temperature, however, might not be the best one in this case. As discussed before, according to literature data^23,24^ and Figure 4, the ideal temperature would be between 305 and 310 °C. Therefore, 310 °C was selected as the treatment temperature for the objectives of the current work.

### Delignification Gravimetry

The samples of wood were delignified as an indirect way to quantify hemicellulose thermodegradation. By the same line of thought used in the DTG deconvolutions, the temperature at which cellulose begins to degrade would cause a drastic increase in the relative amount of lignin in the sample, resulting in a sharp increase in the mass loss caused by delignification. Figure 7a presents the delignification values for fir. It shows a noticeable increase in relative lignin content at 310 °C, supporting the choice of that temperature as the most appropriate for selectively degrading hemicelluloses in fir. Delignification of F315 was not attempted due to how little mass was left after the thermal treatment.

**Figure 7:**
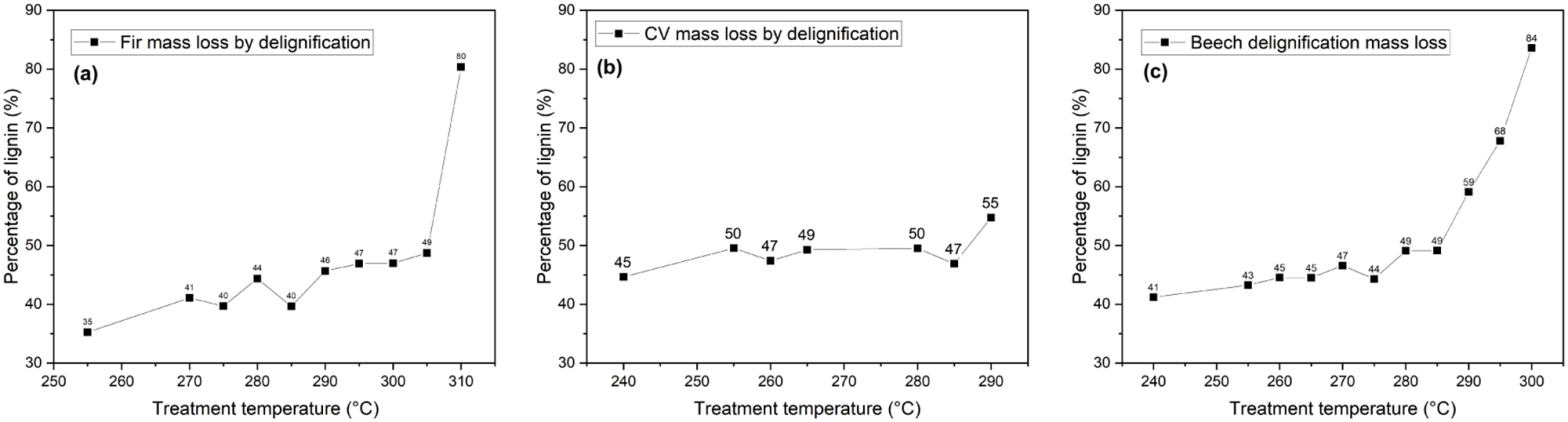
Delignification of (a) fir, (b) CV, and (c) beech samples treated at different temperatures.

Figure 7b represents the delignification results of CV samples, which also support the choice of 290 °C as the ideal temperature for the selective thermal degradation of hemicelluloses in CV. The delignification of CV295 was attempted, but different from all the other delignifications done in this work, which resulted in a fibrous pulp, this sample resulted in a gelatinous material which could not be filtered by usual methods, further suggesting 290 °C to be the ideal temperature for CV.

Figure 7c represents the delignification results of beech. For beech, an unexpected behavior is observed. The increase in lignin mass loss is seen in B280, and not in B275, as expected considering the previous results for this wood. This could be caused by a greater separation between the degradation temperature of the biopolymers in beech (a hardwood).^6^ It is also possible, like it was the case for fir, that the ideal temperature would be between 275 and 280 °C, but further studies are necessary to confirm these hypotheses. Despite the delignification results, as all other results pointed to 275 °C as the ideal temperature to selectively degrade hemicelluloses in beech, B275 was chosen for the preparation of composites.

### Particle Size Selection

FTIR was used to select the best particle size for the preparation of the composites. The objective was to see which particle size would provide the largest amount of exposed −OH for the reaction with NCO. Figure 8 shows the FTIR spectra obtained.

**Figure 8:**
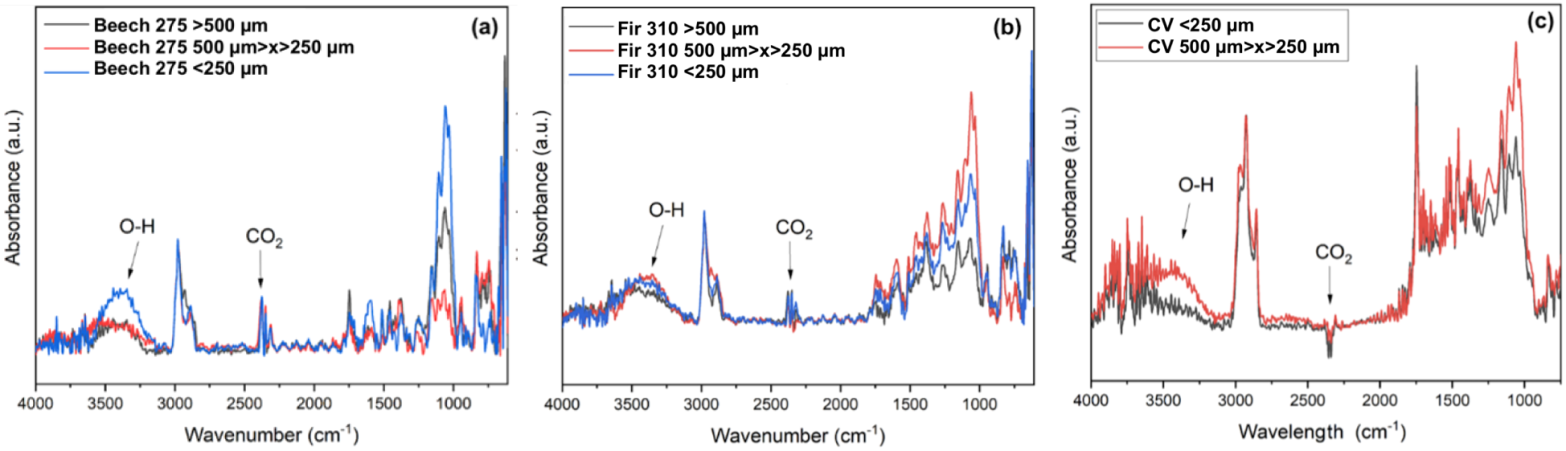
FTIR spectra of (a) beech, (b) fir, and (c) CV in different particle sizes.

Figure 8 shows a clear difference in exposed hydroxyl groups (3400 cm^–1^) for the smallest beech particle size, making it the clear choice for composite preparation. For fir, the difference of the O–H peak is negligible for the different particle sizes, therefore, particles <250 μm were chosen for composite preparation. CV presented anomalous behavior, with a much bigger O–H band for the intermediate size. This would, at first, make it the size of choice for composite preparation, but there are other factors to be considered. As shown in the works of Edoziuno et al. (2025)^25^ and Jaya et al. (2016),^26^ bigger particle sizes lead to more brittle composites, as the particles need bigger voids in the polymeric matrix to house them, making it more fragile. For this reason, and also to keep the uniformity of the composites, particles <250 μm were also chosen for the preparation of CV composites.

### Composites

Based on the results discussed in previous sections, the chosen samples for the preparation of composites were B 275, F 310, and CV 290, with a particle size <250 μm. The composites presented in this work are labeled according to the wood used, whether it was untreated or thermally treated (identifying the temperature used), the percentage of wood used, and its NCO/OH ratio, adding Comp at the beginning to identify them as composites. For example, Comp B 275 2% 0.8 represents a composite prepared with 2 wt % of B 275 and a 0.8 NCO:OH ratio, while Comp F Raw 5% 1.0 is prepared with 5 wt % of untreated fir and a 1.0 NCO:OH ratio. Figure 9 shows a picture of all composites prepared for this work.

**Figure 9:**
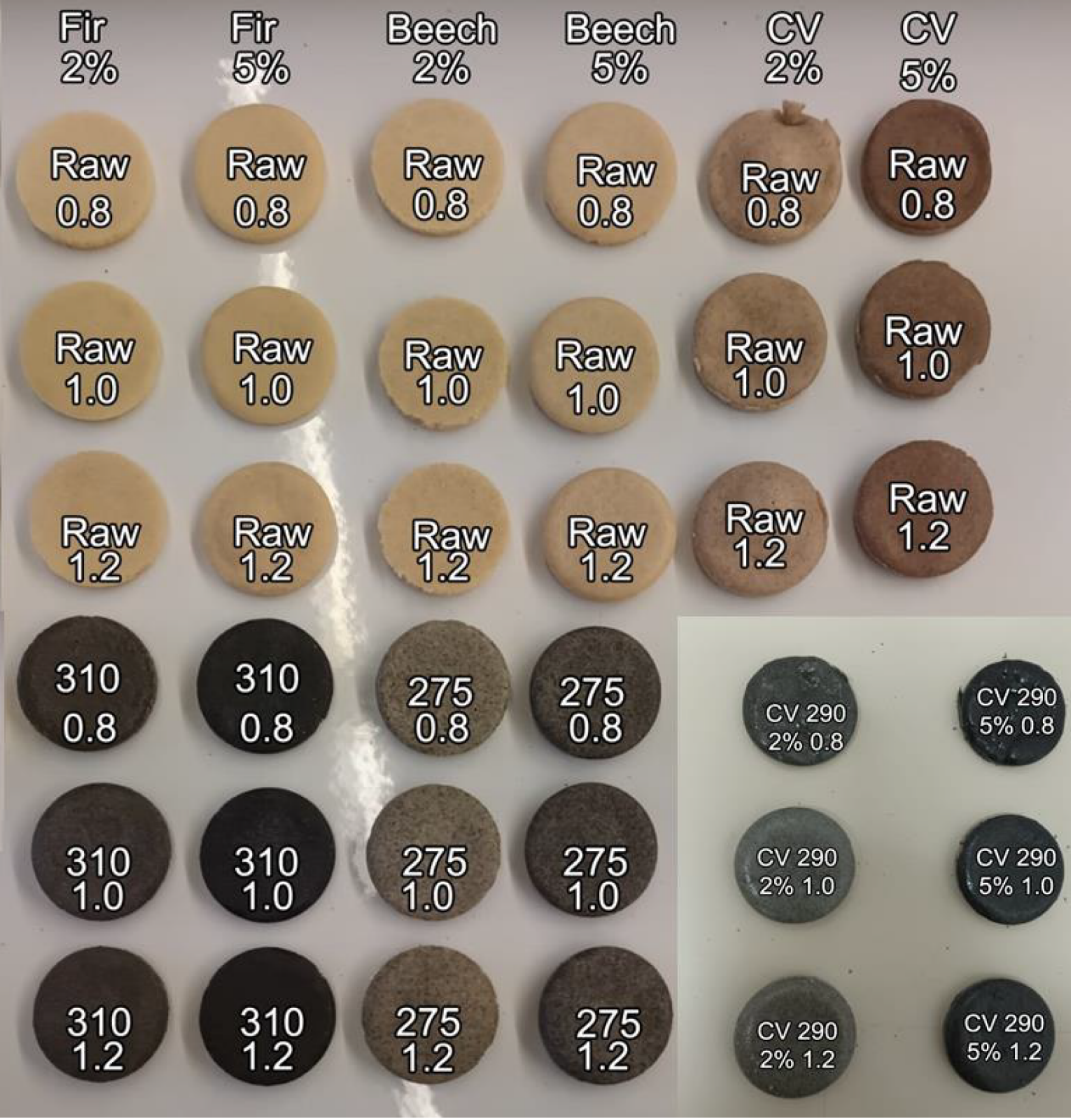
Photographs taken by the authors of composites prepared for this work.

The FTIR spectra of the composites exhibit only minor differences due to their similarity in composition and low reinforcement loading. Indeed, differences in wood species and thermal treatment do not significantly affect the overall chemical profile of the samples. Therefore, representative spectra of composites containing 5 wt % of wood were selected to point at key differences observed (Figure 10), as the higher wood content leads to slightly more accentuated differences. A residual O–H peak seen in all spectra at approximately 3500 cm^–1^ indicates the presence of either unreacted hydroxyl groups in the final product, possibly coming from regions of the wood that were not accessible to the NCO, or trace water absorbed from moisture in the air after composite preparation. This residual O–H signal, however, was smaller in the treated CV composite, possibly indicating a greater availability of hydroxyl groups for the NCO in this sample, which can also be inferred by the relatively bigger C–N peaks observed in these samples compared to composites containing other wood species. Composites containing untreated CV also exhibit bigger C–N peaks, but their residual O–H signals are bigger than the treated samples, indicating that this wood might have a naturally bigger amount of available hydroxyl groups compared to the other wood species both in treated and untreated states.

**Figure 10:**
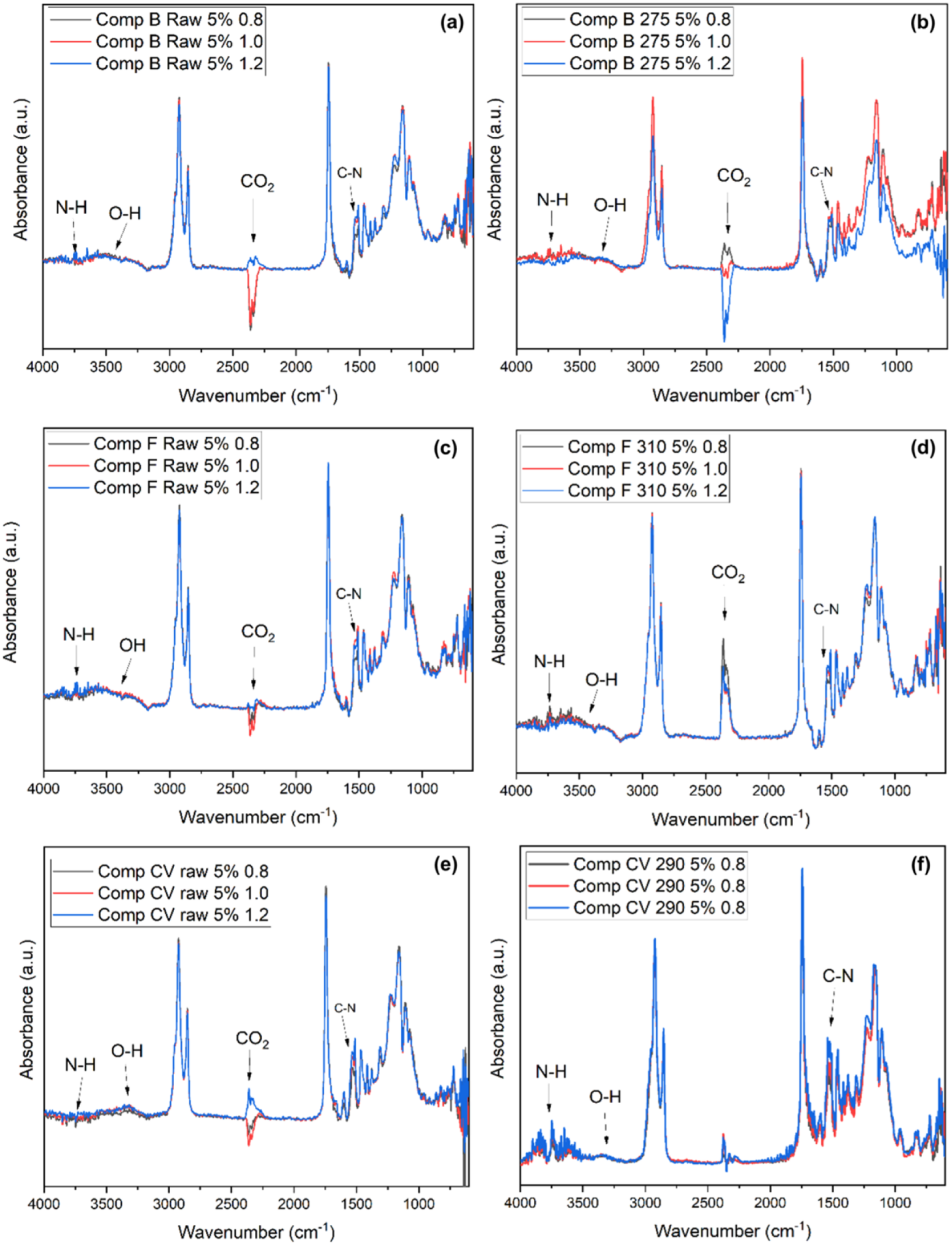
FTIR spectra of composites prepared with 5 wt % of (a) untreated beech and (b) beech treated at 275 °C, (c) untreated fir and (d) fir treated at 310 °C, and (e) untreated CV and (f) CV treated at 290 °C.

The TGA and DTG analyses of all samples also show little difference between the thermal behavior of the samples, as will be seen further ahead, showing that the different NCO:OH ratios and the addition of wood had little impact on the sample’s thermal properties, most likely due to the similar compositions and low reinforcement loading. A point of interest is seen in the DTG curves of the samples with an NCO:OH ratio of 1.0. As seen previously,^5^ only the 1.0 NCO:OH ratio PU exhibits the three typical peaks associated with polyurethane thermal degradation, corresponding to the cleavage of urethane linkages, degradation of hard segments (NCO-rich domains), and degradation of soft segments (polyol-rich domains), while the other NCO:OH ratios exhibit four peaks. The presence of four peaks indicates a more complex structure, with segments of intermediate rigidity. The composites with a 1.0 NCO:OH ratio, though, also presented four degradation peaks, likely caused by the new −OH groups added into the polymer by the addition of the lignocellulosic reinforcement material, as will be seen in Figure 11.

**Figure 11:**
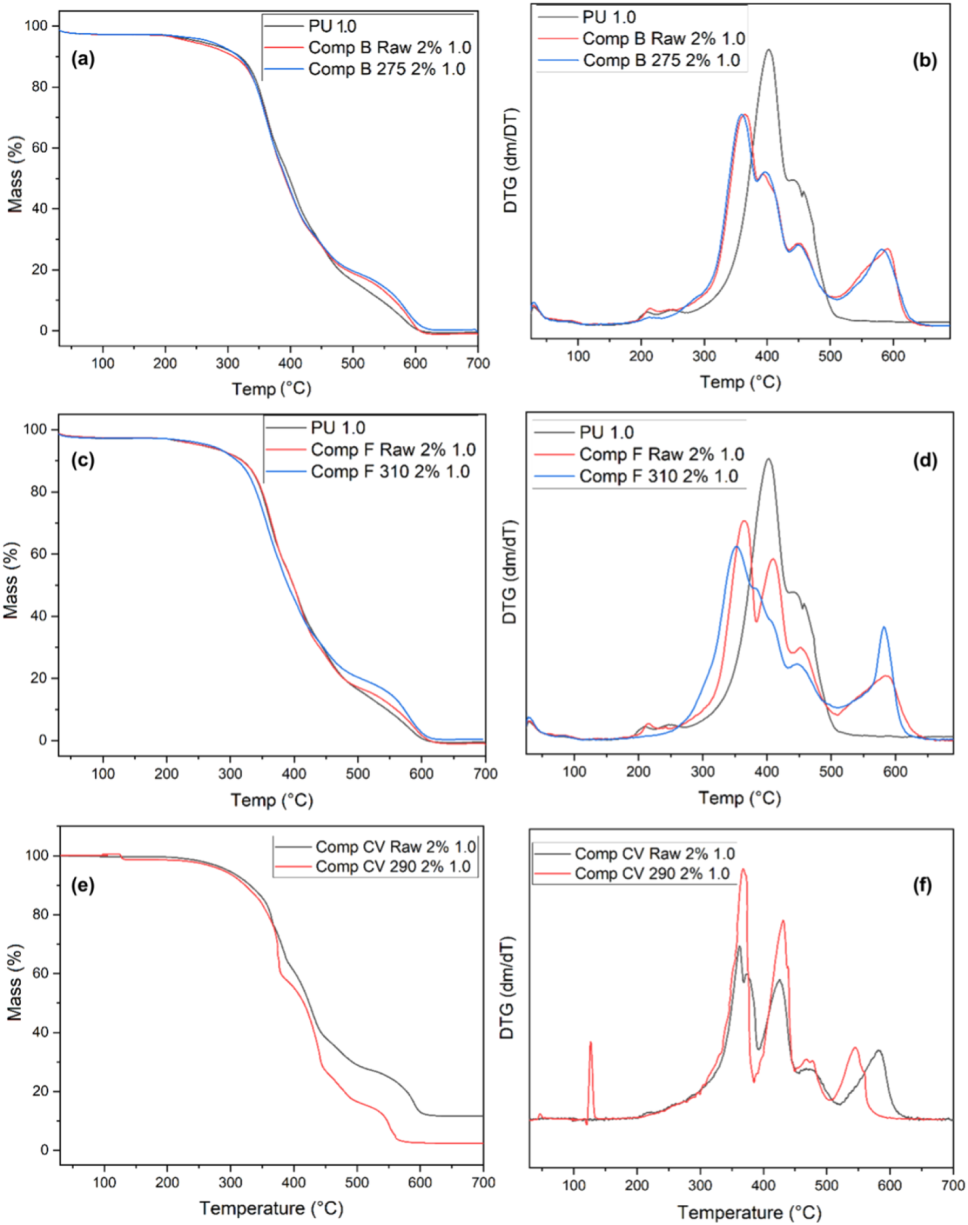
(a) TGA of PU with a NCO:OH ratio of 1.0, and composites prepared with 2 wt % of beech untreated and treated at 275 °C. (b) DTG curves of PU with a NCO:OH ratio of 1.0, and composites prepared with 2 wt % of beech untreated and treated at 275 °C. (c) TGA of PU with a NCO:OH ratio of 1.0, and composites prepared with 2 wt % of fir untreated and treated at 310 °C. (d) DTG curves of PU with a NCO:OH ratio of 1.0, and composites prepared with 2 wt % of fir untreated and treated at 310 °C. (e) TGA of composites prepared with 2 wt % of CV untreated and treated at 290 °C. (f) DTG curves of composites prepared with 2 wt % of CV untreated and treated at 290 °C.

Figures 11a–d show the presence of 4 decomposition steps for beech and fir composites, while the raw CV composite exhibits five steps (Figures 11e and 11f), which can be related to the higher amount of −OH groups discussed in the FTIR results, showing the effect that the addition of raw and treated wood had on the complexity of the polymeric structure. Notably, very little variation is noticed between composites and unreinforced PUs at temperatures lower than 350 °C (Figures 11a–f). It can be observed in Figures 11a–d that the composites prepared with thermally treated wood exhibit a higher weight % between 500 °C and 600 °C. This can be attributed to the fact that after the thermal treatment is applied, the wood had already been partially thermally degraded, leaving a higher concentration of the more thermally resistant components in the biomass. Interestingly, CV composites do not follow that same behavior.

It should also be emphasized that even at low filler loadings (2 wt %), thermally treated wood may influence local cure behavior, cross-link density, and free volume, particularly due to OH-rich interfaces altering the effective NCO:OH ratio locally. Indeed, the thermal treatment applied is expected to decrease the number of hydroxyl groups on the surface of the wood, directly affecting covalent bonding between the polymer matrix and reinforcement.^13–15^ With less covalent interactions, treated reinforcement is more susceptible to thermal degradation during TGA than nontreated reinforcement. This effect can be clearly observed for composites prepared with fir and carvoeiro (Figure 11c–f), for which a lower weight % is obtained between 300 °C and 400 °C for composites made with treated wood in comparison to those made with nontreated wood.

A comprehensive list of thermal properties obtained from the numerous TGAs and DTGs performed for this work can be found in Table S1, where *T*_onset_ means the temperature at which the thermal degradation begins, *T*_5_ is the temperature at which the sample achieves 5 wt % loss of its original mass, and *T*_10_ is the temperature at which the sample achieves 10 wt % loss of its original mass. *T*_onset_ for the unreinforced PUs shows little variation regardless of the NCO:OH ratio, with a highest *T*_onset_ of 339.2 °C for PU 0.8 and a lowest *T*_onset_ of 332.3 °C for PU 1.0 (Table S2). *T*_5_ and *T*_10_, however, consistently decrease with higher NCO:OH ratios for unreinforced PUs, revealing a faster degradation rate with more soft segments/unreacted polyols. No striking differences or particular thermal degradation trends that hold true for all samples can be established for the composites prepared in this work. It is possible that the loadings of 2 and 5 wt % employed do not result in significant changes in local cure behavior and the matrix reinforcement interface to the extent of affecting the overall thermal stability of the bulk composites in an undisputed consistent manner.

In order to investigate the impact of thermally treated woods on the water sorption behavior of PU composites, DVS experiments were conducted on unreinforced PUs of different NCO:OH ratios and their corresponding composites containing 2 wt % of untreated and thermally treated beech and fir. These results are summarized in Table 2, while full sorption and desorption curves are provided in Figure S1. The DVS analysis of CV composites could not be accomplished due to a major failure of the equipment used. Although their water sorption behavior is expected to follow those observed and discussed herein for beech and fir, experimental DVS data should be collected in the future to confirm the expected trends.

**Table 2:** Water Uptake at 95% Relative Humidity from DVS Experiments for PUs of Different NCO:OH Ratios and Their Corresponding Composites Containing 2 wt % of Untreated and Thermally Treated Beech and Fir

Entry	NCO:OH ratio	Reinforcement (2 wt %)	Treatment temperature (°C)	Water uptake at 95% relative humidity (%)
**1**	0.8	-	-	1.80
**2**	1.0	-	-	1.60
**3**	1.2	-	-	1.35
**4**	0.8	beech	-	2.24
**5**	1.0	beech	-	2.21
**6**	1.2	beech	-	2.19
**7**	0.8	beech	275	14.19
**8**	1.0	beech	275	15.86
**9**	1.2	beech	275	14.43
**10**	0.8	fir	-	2.01
**11**	1.0	fir	-	1.75
**12**	1.2	fir	-	1.91
**13**	0.8	fir	310	16.22
**14**	1.0	fir	310	15.94
**15**	1.2	fir	310	18.57

As can be seen in Table 2, **entries 1–3**, as well as in Figure S1, unreinforced PUs exhibit similar water sorption behavior regardless of their NCO:OH ratios, with water uptake at 95% relative humidity ranging from 1.80% to 1.35%. The relatively low water uptake numbers reveal the hydrophobic character of the PU matrix. The introduction of 2 wt % of untreated beech (Table 2, **entries 4–6**) and untreated fir (Table 2, **entries 10–12**) did not significantly affect the water sorption behavior of the samples in comparison to the unreinforced PUs (Table 2, **entries 1–3**), with water uptake at 95% relative humidity ranging from 1.75% (Table 2, **entry 11**) to 2.24% (Table 2, **entry 4**). No specific correlation can be made between the water uptake at 95% relative humidity, the NCO:OH ratio, or untreated wood species. It is understood that the wood particles added are fully enveloped by the hydrophobic matrix, therefore not changing the water sorption behavior.

When the wood particles added to the PU matrix undergo a thermal treatment to remove hemicelluloses, a great change is observed in the water sorption behavior of the composites (Table 2, **entries 7–9** and **13–15**), with water uptake at 95% relative humidity ranging from 14.19% (Table 2, **entry 7**) to 18.57% (Table 2, **entry 15**). The increase in water uptake at 95% relative humidity noticed with the addition of thermally treated wood seems independent of the wood species. This increase cannot be explained by the polarity of the samples, as it is well-known that thermally treated woods are more hydrophobic than untreated woods.^6,27^

There are two possible factors potentially contributing to the observed increase in water sorption behavior for composites prepared with thermally treated woods. First, it has been demonstrated that higher treatment temperatures can increase the wood sample porosity, allowing water condensation inside its pore structure and resulting in a sorption behavior similar to hygroscopic materials.^27,28^ The density of beech and fir composites prepared with treated and untreated wood particles at 2 wt % loading has been determined, and the results are presented in Table S3. It can be clearly seen that the composites made with thermally treated wood are significantly less dense than those prepared with untreated wood. Indeed, the density of untreated beech composites ranges from 0.41 g/mL to 0.50 g/mL, while that of thermally treated beech composites ranges from 0.19 g/mL to 0.26 g/mL (Table S3). Likewise, the density of fir composites goes from 0.33 to 0.44 g/mL when untreated wood is used, to 0.23–0.29 g/mL when thermally treated wood is used (Table S3). These results corroborate the idea that a more porous structure is obtained when thermally treated wood is used as reinforcement. However, the contribution from this factor alone can be hampered by the fact that the wood particles are completely enveloped by the hydrophobic polymeric matrix, as discussed earlier in the text, and therefore are not directly exposed to the moisture-saturated environment during the DVS experiments.

As a working hypothesis, the second possible contributing factor for the increased water uptake lies in the potential formation of mesopores, triggered by the higher porosity of the thermally treated woods. The water sorption mechanism in mesopores starts with the initial formation of a molecular monolayer of water that covers the internal surface of the material, followed by the filling of micropores (<2 nm) in the structure. This initial step is likely analogous to the mechanism occurring during water sorption in the unreinforced PUs and in the composites containing 2 wt % of untreated wood. After micropore filling, a phenomenon known as capillary condensation happens in mesopores (2–50 nm) and, to a lower extent, in smaller macropores (>50 nm). During capillary condensation, liquid water condenses inside those larger pores,^29^ leading to a much higher gain in mass for these samples in comparison to the molecular monolayers of water and micropore fillings observed for unreinforced PUs and composites containing 2 wt % of untreated wood. The results imply that the addition of thermally treated wood leads to the formation of a bigger quantity of mesopores in the structure. It is worth noting that although the proposed explanation linking increased water sorption to mesopore formation and capillary condensation is reasonable and supported by the literature, it remains speculative without direct microstructural evidence.

## Conclusions

The three wood species investigated in this study, namely, beech, fir, and carvoeiro (CV), were first analyzed by density measurements, which confirmed that beech (a hardwood) is denser than fir (a softwood). CV exhibits a density nearly twice as that of beech. Upon a detailed and systematic assessment of the impact of thermal treatments at different temperatures on the composition of treated woods, ideal treatment temperatures were established for each wood investigated. The results obtained were consistent and aligned with literature data. The established ideal temperatures for selective hemicellulose degradation were 275 °C for beech, 290 °C for CV, and 310 °C for fir. During those experiments, the analysis of deconvoluted DTG curves proved to be a practical and reliable method for the determination of wood composition. The thermal profiles obtained for beech and fir are in accordance with the nature of hemicelluloses of both hard- and softwoods, with softwood degrading at a higher temperature. For CV, despite it being a hardwood, its mass loss values during thermal treatment are similar to fir (a softwood), indicating a higher natural thermal stability possibly associated with an evolution to survive in its biome (Brazilian Cerrado). Based on FTIR data, a particle size <250 μm was selected for the preparation of composites. FTIR and TGA data revealed that the composites had similar chemical and thermal properties, regardless of the NCO:OH ratio, wood species, loading, or thermal treatment. DVS experiments revealed a significant increase in water uptake for composites reinforced with thermally treated woods. It is hypothesized that the observed increase could be associated with the formation of a mesopore structure in the polymeric matrix, possibly triggered by the higher porosity of thermally treated wood in comparison to untreated wood. In order to fully understand the sorption mechanisms taking place in thermally treated wood biomass, supporting data on scanning electron microscopy (SEM) as well as surface area/porosity studies via Brunauer–Emmett–Teller (BET) measurements is necessary and should be pursued in the future. Another limitation of the results presented in this study is the absence of DVS data for CV composites. Although their water sorption behavior is expected to follow those observed herein for beech and fir, experimental DVS data should be collected in the future to confirm the expected trends.

## Supplementary Material



## Notes

Table S1: % area under DTG deconvoluted curves for beech treated at different temperatures; Table S2: thermal properties of all PUs and composites produced in this work; and Figure S1: vapor sorption and desorption curves of (a) PU with a 0.8 NCO:OH ratio and corresponding treated and untreated beech composites, (b) PU with a 1.0 NCO:OH ratio and corresponding treated and untreated beech composites, (c) PU with a 1.2 NCO:OH ratio and corresponding treated and untreated beech composites, (d) PU with a 0.8 NCO:OH ratio and corresponding treated and untreated fir composites,(e) PU with a 1.0 NCO:OH ratio and corresponding treated and untreated fir composites, and (f) PU with a 1.2 NCO:OH ratio and corresponding treated and untreated fir composites (PDF)
