# Supplementary material for: Bio-based Polyurethane Composites from Macaúba Kernel Oil: Part 2, Wood Thermal Treatment and Composite Water Sorption Properties
Source: ACS Omega. 2026 Jul 10;11(29):44243–54. doi: 10.1021/acsomega.6c04518 (PMC13425306; doi:10.1021/acsomega.6c04518)
Supplement: Supplementary file 1 [file ao6c04518_si_001.pdf]

Bio-based Polyurethane Composites from Macauba Kernel Oil: Part 2, Wood Thermal Treatment and Composite Water Sorption Properties

Rodolfo Andrade Breves,[a,b] Roseany de Vasconcelos Vieira Lopes,[c] Rafael Lopes Quirino,[d,*] Baptiste Colin,[b] Anelie Petrissans,[b] Maria José Araújo Sales,[a] Mathieu Petrissans[b]

[a] *University of Brasília, Chemistry Institute, LabPolN, Campus Darcy Ribeiro, 70910-000, Brasilia-DF, Brazil.*

[b] *IUT Hubert Currien, LERMAB, Université de Lorraine, 88000, Epinal, France.*

[c] *University of Brasília, Campus Gama, 72429-005, Gama-DF, Brazil.*

[d] *Center for Advanced Materials Science, Department of Biochemistry, Chemistry and Physics, Georgia Southern University, 30460, Statesboro, GA, USA.*

** Correspondence to: rquirino@georgiasouthern.edu*

Supplementary Information

Table S1. % area under DTG deconvoluted curves for beech treated at different temperatures.

Treatment temperature (˚C)	Water (%)	Hemicelluloses (%)	Cellulose (%)	Lignin (%)	Others (%)
-	1.6	25.8	44.8	14.4	13.4
260	2.3	20.2	37.4	13.1	27.0
270	1.8	7.6	45.0	42.2	3.4

Table S2. Thermal properties of all PUs and composites produced in this work.

Sample	T_{onset} (°C)	T_5 (°C)	T_{10} (°C)
**PUs**[a]			
PU 0.8	339.2	273.3	326.8
PU 1.0	332.3	254.6	314.3
PU 1.2	332.7	249.6	313.8
**Beech 2%**			
Comp B Raw 2% 0.8	335.0	242.2	318.9
Comp B 275 2% 0.8	330.1	269.2	319.6
Comp B Raw 2% 1.0	331.6	242.3	311.8
Comp B 275 2% 1.0	317.6	271.8	319.3
Comp B Raw 2% 1.2	330.1	254.5	313.2
Comp B 275 2% 1.2	322.8	266.8	311.5
**Fir 2%**			
Comp F Raw 2% 0.8	334.4	242.2	316.8
Comp F 310 2% 0.8	332.3	264.3	322.0
Comp F Raw 2% 1.0	334.3	257.0	319.3
Comp F 310 2% 1.0	321.0	266.9	311.5
Comp F Raw 2% 1.2	329.4	244.7	303.9
Comp F 310 2% 1.2	319.9	271.9	311.6
**CV 2%**			
Comp CV Raw 2% 0.8	343.8	305.1	334.5
Comp CV 290 2% 0.8	325.5	314.3	341.8
Comp CV Raw 2% 1.0	336.5	269.4	331.1
Comp CV 290 2% 1.0	343.9	288.7	323.1
Comp CV Raw 2% 1.2	329.1	299.5	322.6
Comp CV 290 2% 1.2	318.9	293.9	317.8
**Beech 5%**			
Comp B Raw 5% 0.8	326.1	259.6	304.1
Comp B 275 5% 0.8	316.9	266.8	304.3
Comp B Raw 5% 1.0	322.1	254.4	301.6
Comp B 275 5% 1.0	317.6	259.5	301.6
Comp B Raw 5% 1.2	317.3	262.0	299.2
Comp B 275 5% 1.2	317.7	269.3	309.2
**Fir 5%**			
Comp F Raw 5% 0.8	322.3	247.1	294.1
Comp F 310 5% 0.8	319.1	281.8	314.3
Comp F Raw 5% 1.0	315.0	264.4	301.7
Comp F 310 5% 1.0	319.1	281.8	314.3
Comp F Raw 5% 1.2	319.9	264.4	306.7
Comp F 310 5% 1.2	317.1	281.9	312.1
**CV 5%**			
Comp CV Raw 5% 0.8	343.2	294.9	318.4
Comp CV 290 5% 0.8	337.6	304.9	336.5
Comp CV Raw 5% 1.0	328.2	293.8	321.1
Comp CV 290 5% 1.0	332.1	304.7	326.4
Comp CV Raw 5% 1.2	326.1	299.5	232.6
Comp CV 290 5% 1.2	320.0	303.4	326.1

[a] From reference [5].

Table S3. Density of beech and fir composites prepared with treated and untreated wood particles at 2 wt.% loading

Sample	Density (g/mL)
Comp B Raw 2% 0.8	0.41
Comp B Raw 2% 1.0	0.45
Comp B Raw 2% 1.2	0.50
Comp B 275 2% 0.8	0.26
Comp B 275 2% 1.0	0.19
Comp B 275 2% 1.2	0.24
Comp F Raw 2% 0.8	0.33
Comp F Raw 2% 1.0	0.44
Comp F Raw 2% 1.2	0.40
Comp F 310 2% 0.8	0.29
Comp F 310 2% 1.0	0.23
Comp F 310 2% 1.2	0.26

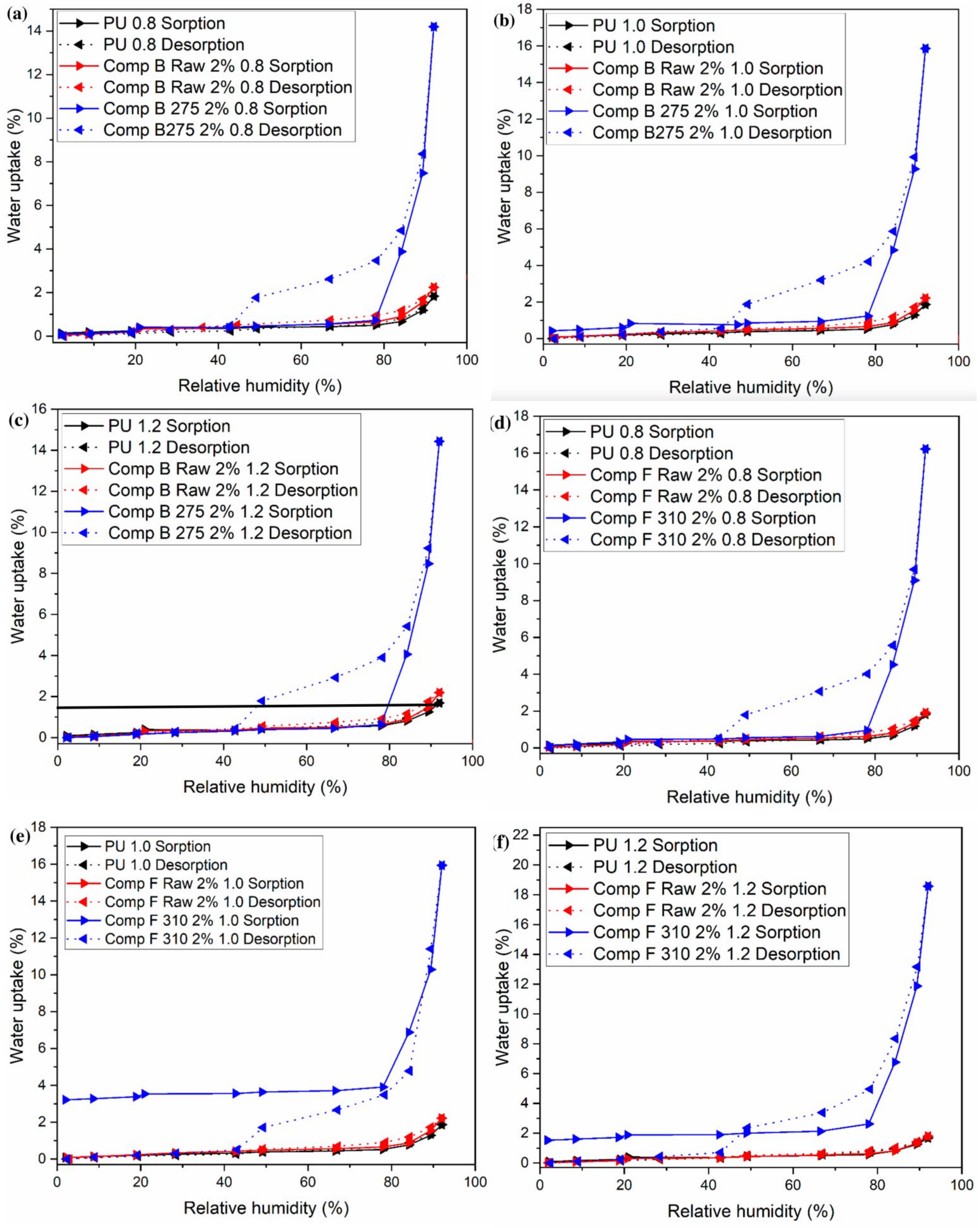

Figure S1. Vapor sorption and desorption curves of **(a)** PU with 0.8 NCO:OH ratio and corresponding treated and untreated beech composites, **(b)** PU with 1.0 NCO:OH ratio and corresponding treated and untreated

beech composites, **(c)** PU with 1.2 NCO:OH ratio and corresponding treated and untreated beech composites, **(d)** PU with 0.8 NCO:OH ratio and corresponding treated and untreated fir composites, **(e)** PU with 1.0 NCO:OH ratio and corresponding treated and untreated fir composites, **(f)** PU with 1.2 NCO:OH ratio and corresponding treated and untreated fir composites.
